# Kenyan palliative care providers’ and leaders’ perceptions of palliative care research needs and support to facilitate rigorous research

**DOI:** 10.1186/s12904-023-01199-0

**Published:** 2023-09-12

**Authors:** K. B. Cartmell, E. A. Doherty, N. Gikaara, Z. Ali, S. Qanungo, E. S. Melikam, R. A. Powell

**Affiliations:** 1 Department of Public Health Sciences, College of Behavioral, Social and Health Sciences, Clemson University, Clemson, SC USA; 2 Department of Medicine, Aga Khan University Hospital, Nairobi, Kenya; 3 Kenyan Hospice and Palliative Care Association, Nairobi, Kenya; 4 College of Nursing, Medical University of South Carolina, Charleston, SC USA; 5 Department of Primary Care and Public Health, Faculty of Medicine, Imperial College London, London, England; 6 Ethnicity and Health Unit, NIHR Applied Research Collaboration Northwest London, London, England; 7 MWAPO Health Development Group, Nairobi, Kenya

**Keywords:** Palliative care, Hospice, Research capacity, Qualitative research, Focus groups

## Abstract

**Background:**

Palliative care (PC) can reduce symptom distress and improve quality of life for patients and their families experiencing life-threatening illness. While the need for PC in Kenya is high, PC service delivery and research is limited. Qualitative research is needed to explore potential areas for PC research and support needed to enable that research. This insight is critical for informing a national PC research agenda and mobilizing limited resources for conducting rigorous PC research in Kenya.

**Objectives:**

To explore perceptions of priority areas for PC research and support needed to facilitate rigorous research from the perspective of Kenyan PC providers and leaders.

**Methods:**

Focus groups (FGs) were conducted in November and December of 2018 using a semi-structured interview guide. FGs were audio-recorded, transcribed, and analyzed using a thematic content analysis approach.

**Results:**

Three FGs were conducted (*n* = 22 participants). Ten themes related to PC research emerged, including research on: 1) beliefs about death, disease, and treatment to inform PC; 2) awareness about PC, 3) integration of PC within the health system; 4) understanding caregiver experiences and needs; 5) community health volunteers (CHVs) and volunteer programs; 6) evaluation of costs and benefits of PC; 7) treatment approaches, including complementary and alternative medicine (CAM) and advanced diagnostics at end of life; 8) other suggestions for research, 9) populations in need of PC research; and 10) resources for enabling research.

**Conclusions:**

Kenyan PC providers and leaders identified key areas requiring increased scientific inquiry and critical resources needed to enable this research. These findings can help to focus future PC research in Kenya and encourage funding agencies to prioritize the issues identified.

## Background

In Kenya, need for palliative care (PC) is overwhelming, as more people are diagnosed with chronic conditions, alongside already high levels of acute infectious diseases such as malaria and tuberculosis. Approximately 800,000 Kenyans require PC annually, but only about 14,552 receive these services [1]. Much of the need for PC in Kenya is driven by high levels of HIV disease and cancer, both of which have a high symptom burden. In 2021 the HIV prevalence rate in Kenya was 4.0% among adults ages 15–49 [2]. Additionally, the incidence of cancer in Kenya increased from 37,000 in 2012 to 47,887 in 2018, with cancer now ranking as the third leading cause of death after infectious and cardiovascular diseases [3, 4]. Despite the growing need for PC in Kenya, PC remains inaccessible for most patients, particularly in rural areas.

While PC remains a relatively new discipline in Kenya and other African countries, [5] Kenya is poised to make significant progress in PC service delivery [6]. Kenya is one of the first African nations to formally integrate PC into healthcare policy and national cancer plans. Additionally, Kenya has a well-established national organization, the Kenyan Hospices and Palliative Care Association (KEHPCA), that works to increase PC access in Kenya using a multisectoral approach, that brings together healthcare providers, national and local governments, development partners, local communities, and patients and families in need of PC [7]. Kenya’s national focus and evolving infrastructure provides a unique opportunity to build a PC system in Kenya that can serve as a model for other African countries.

Unfortunately, most evidence to inform PC delivery has been developed and evaluated in high-resource countries like the US and UK, [8] with just over 1% of scientific PC literature originating from low-resource countries [6]. Findings from high-resource countries may not always be relevant to the unique aspects of health, finance and culture surrounding PC provision in Africa. Lack of local research activity can be partially attributed to limited resources, competition with other health priorities, and lack of infrastructure to connect the limited number of researchers in this field. Moreover, international PC research in Africa is often characterized by short-term, project-specific commitment, inadequate financing, and over-dependency upon key individuals [9]. The current study explores perceptions of needed research and the support required to carry out this research among PC healthcare providers, staff and thought leaders in Kenya. These insights can inform a national palliative research agenda, foster research collaboration, and inform use of limited resources.

## Methods

### Study aim and design

A qualitative thematic content analysis was conducted with Kenyan PC providers and leaders to explore perceptions of PC research needs and support required to facilitate high quality PC research in Kenya.

### Study setting

The study was conducted in Nairobi, Kenya, in partnership with KEHPCA and the Medical University of South Carolina in conjunction with the 2018 KEHPCA National PC Conference which hosted PC providers and leaders from across Kenya and neighboring countries.

### Participants and recruitment

Recruitment for the focus groups was carried out during the 3-day KEPHCA conference via announcement at the conference, emails, and flyers. Anyone who was a hospice or palliative care professional was eligible for the study. Participants included a broad range of palliative and hospice physicians, nurses, medical assistants, social workers, national thought leaders, and other professionals who attended the conference.

### Data collection

A semi-structured interview guide was developed by our research team for use in the focus groups (FGs). The guide queried: perceptions about PC in Kenya, including strengths, weaknesses and needed improvements; challenges faced in delivering PC, and type of research needed by topic area (e.g. assessment tools, bereavement, caregivers), by symptoms and diagnoses (e.g. cancer, HIV/AIDS), and by populations (e.g. children, older people, prisoners), prior experiences and interest conducting research, and support that would help an individual and their organization to conduct research. Before FGs, the voluntary nature of the study was explained to participants, and they were informed that their participation in the FG indicated their informed consent. FGs were facilitated by a researcher with experience conducting FGs and familiarity with Kenyan PC professionals. FGs were audio-recorded, transcribed verbatim by a research team member, and checked for accuracy by the study PI (KC).

### Data analysis

An inductive thematic content analysis of FG transcripts was performed, [10] with the aim of understanding PC research priorities as perceived by participants. Coding was conducted by two researchers who independently coded and analyzed data. Each researcher initially read the transcripts to create their own independent codebooks, which they compared and discussed to create the initial study codebook. As new transcripts were analyzed, the codebook was refined to create additional codes or merge codes to fit the data. Codes were compared, grouped, and aggregated into broader thematic categories. Quotes were selected to illustrate findings. Impressions were discussed between researchers, with discrepancies resolved between the two researchers by reviewing representative quotes and going back to the original transcripts as needed to reach consensus. While it is possible that 100% saturation was not reached with 3 focus groups, our research team was confident that we were able to identify most (if not all) themes, along with detailed insights into the most common themes [11]. A final report of themes and sub-themes was generated. Analysis was conducted in NVivo 11 (QSR International Pty Ltd., Doncaster, Victoria, Australia) and manually, with an audit trail maintained to record the analytic process.

## Results

Data were collected during three FGs, with 22 participants (12, 6, and 4 per FG, respectively). Nearly all participants were of African ancestry, with participant ages ranging from 38–79 and approximately two-thirds being females. Each FG lasted between 1 to 1.5 h. Participants shared insight about priority areas for research and factors that influence PC research, resulting in ten thematic priority areas and 32 topics of interest. An overview of these themes and topics is presented in Table 1.

**Table 1:** Areas of PC research interest in Kenya

Priority Areas:		Topics of interest for research:
1	Beliefs about death, disease, and treatment that can inform PC	• Patient culture and provision of culturally competent PC services • Provider beliefs and care transition
2	Awareness of PC	• Provider awareness about PC • Public awareness about PC • Implementation of evidence-based education initiatives
3	Integration of PC within the health system	• Integration within medical system • PC education and training • Government support at national and county level
4	Understanding caregiver experiences and needs	• Family and paid caregiver experiences and needs, including bereavement support • PC provider experiences and needs
5	Community health volunteers (CHV) and volunteer programs	• Traditional CHV and volunteer programs • Opportunities for additional community partnerships
6	Evaluation of costs and benefits of PC	• Cost of providing basic PC • Comparison of cost–benefit of different PC models (e.g. home, hospital, hospice) • Use of this information for advocacy
7	Utility of treatment approaches, Including complementary and alternative Medicine (CAM) and use of advanced diagnostics at end of life	• Are these practices (e.g. yoga, medication, herbal remedies) effective • Can they be replicated for use in other settings • Evaluation of benefits of extensive diagnostic tests and treatments when a cure is not possible
8	Other suggestions for research	• Evaluation of strategies for PC advocacy • Quality of care and process evaluation • Patient-centered care • Effect of disclosure of terminal disease status on patient • Spirituality in PC • Effect of PC on quality of life • Epidemiology research
9	Populations in need of PC research	• Pediatric PC models and new approaches such as child-life support services • Factors that influence care delivery in remote settings and optimal models for remote PC delivery • Expansion of PC research beyond HIV and cancer to populations often overlooked for services
10	Resources for enabling research	• Human resources (staffing, training, retention) • Enhancement of data collection infrastructure • Cancer registry • Additional sustainable funding

### Beliefs about death, disease and treatment that can inform PC

Participants expressed a desire to conduct research to characterize cultural beliefs surrounding death, disease, and treatment among Kenya’s diverse population, to enhance intervention effectiveness and facilitate tailoring of interventions to meet its diverse needs. Participants identified superstitions surrounding death and disease as barriers to receipt of PC and compliance with care recommendations. One described:

“We have different cultures in this country; we have 47 tribes, 48 now; people who speak different languages…Some take treatment to be taboo. They don’t want certain things done cause of this and that.”

Reflecting on misunderstanding that could arise when patients’ unique cultural beliefs went unrecognized, another participant said:

“I may want to believe looking at her, she may have accepted it’s a disease but … I find she is pulled down by the cultural belief that it is not a disease, somebody has caused it, or she did something wrong, that is why she is getting the suffering as punishment.”

Participants also commented on the need for cultural understanding in provision of bereavement care among Kenya’s diverse population:

“We deal with so many patients from different religions and different tribes… so if we are to introduce the concept of bereavement support from the literature, it has to be contextualized to the locals.”

Thus, understanding patients’ diverse cultural beliefs about death, disease and treatment was a research priority.

Participants also identified a need to study providers’ cultural beliefs that influence patient use of, and transition from, curative to PC. Participants commented on medical providers’ resistance when transferring patients from curative to PC, attributing this resistance to providers belief in their duty to continue attempting life-saving measures. One participant described the internal conflict:

“It’s very difficult for people to make that decision because culturally you feel you are neglecting, or I will look like I will not be blessed.”

Provider resistance to transfer patients to PC was also attributed to cultural stigmas surrounding pain medications. One participant described how cultural beliefs about pain medication prevented collaboration with palliative providers, stating:

“They have operated on a patient and found cancer all over the abdomen, there is nothing else they can do and instead of calling a PC provider some of them still have the fear of morphine.”

Contextual understanding of beliefs about PC within the broader medical community was recognized as a key area requiring further study.

### Awareness of PC

Participants identified a need to explore awareness about PC among medical providers and the community, with a focus on gathering input from vulnerable communities. They attributed resistance of providers and patients towards PC to a common misconception that PC pertains only to end-of-life care. One participant described:

“There is still a lot of stigma when you talk about PC and the reason is people have associated PC with end-of-life care and its entrenched in their minds.”

Another participant added:

“One of the biggest challenges is the public’s perception of PC. Unless the public understands what it is and accepts what it is, it’s very hard for them to see the sites (to get palliative services).”

Conceptualizations of PC were seen by participants as limiting provider collaboration and patient care acceptance, creating discrepancy between known need and lower than expected receptivity to PC in Kenya. Participants explained that studies to understand provider and community beliefs about PC can inform implementation and evaluation of evidence-based education.

### Integration of PC within the health system

Participants expressed a need to evaluate integration of PC within Kenya’s health system. While recognizing increased integration of PC within Kenya’s medical system in recent years, they noted a need for expansion into care settings outside of hospitals and into rural regions. One participant explained:

“For a hospital, we still have not been able to go to more levels…the health center, the dispensary. We still have a lot of work to do.”

Another described:

“I think we are on the right track though, as we have said, the centers are all in the urban [areas].”

Other participants explained that while PC has officially been added as a service in rural counties, there is lack of clear protocols for service delivery and data collection to support PC delivery. Documentation of the level of regional integration was seen as necessary to illuminate current successes and gaps and guide further efforts.

Participants recommended an important aspect of assessing health system integration would be to evaluate the impact of a recent large-scale PC training program for healthcare providers delivered nationally. This was given priority due to concerns about whether those who had been trained were still working in PC. As one participant described:

“Where are these people [following training]? What impact has it created to the communities wherever they are? What level of leadership are they in wherever they are working?”

Participants also placed importance on understanding the attitudes of policymakers and health officers, particularly considering the decentralization of governance and the restructuring of healthcare decision-making in Kenya, giving greater responsibility to county governments.

Participants reported increased national support for PC in the previous years, while noting high variation at the county level. One participant commented:

“Since devolution, it’s a challenge because we now have health committees, and it depends how the county views it. Like where I work, it’s a big challenge.”

Participants described that support should be evaluated across levels of government. Morphine availability was described as one policy area highly salient for PC providers. They described morphine distribution as a “thorny issue” due to limitations of prescribers, cost, and a general fear of morphine. Research in this area was seen as necessary, particularly as policies seeking to increase morphine availability are implemented.

### Understanding caregiver experiences and needs

Participants recognized the need for research on caregiver experiences and burden among family caregivers, paid home care providers, and healthcare providers who care for palliative patients. One participant said:

“You meet this young man who finished high school. The grandfather is alive for the third year, the carer won’t go to college, won’t go dating, won’t go visiting, won’t laugh, won’t smile and it’s like everybody expects him to be stuck with the patient and that is the case for spouses. That is the case for those who are employed, you know even house help who have got caring roles.”

Participants further described a need for exploration around best practices for providing caregivers with bereavement support. They described uncertainty about what is the right amount and timing of bereavement support, and that this preference may vary for different people. One participant remarked that Kenya has a variety of people from different tribes and cultural backgrounds, and that it may be best to: “ask caregivers what they would like and what might help them, and in that way if someone is from one culture, they might request one thing rather than just giving them the service which could be offensive or which could be hurtful.”

Interest in the caregiving experience extended to a desire to study the caregiving experience of palliative healthcare providers. A participant reflected: “PC is very draining. So maybe an evaluation of burn-out.” Participants voiced desire to gain greater understanding of the psychological, social, developmental, and economic impact of caretaking, as a means of identifying the needs and supports for these groups.

### CHVs and volunteer programs

Participants emphasized the important role of CHVs as the “foot soldiers” providing PC in Kenya, suggesting the necessity to further assess the impact of CHV networks. In Kenya, CHVs support delivery of community health services implemented through community health units. They are selected at the community level and trained to provide common prevention and care services. Responsibilities include home visitation, delivery of important health promotion messages, care for common illnesses, and establishment of protocols for delivery of various community health programs [12]. CHVs are also common used in Africa to help with delivery of palliative care services, with roles including direct patient assistance, providing psychosocial / spiritual support, and assisting patients’ families [13].

While CHVs were recognized as being valuable for increasing community contact and continuity of care, several barriers to utilizing CHVs to deliver PC were noted, namely the small size of reimbursement and CHV attitudes toward PC. One participant explained:

“CHVs are a point of follow-up … but what I realized is that some NGOs supporting these things, they somehow give them tips, so at times we train them and we tell them to do the follow-up and due to the fact that there is nothing we are doing to support them, it’s becoming quite difficult for them to do the follow-ups.”

Participants were also interested in examining how community partnerships could expand palliative support to a broader range of patients. One described challenges faced by children accessing palliative support in schools. He explained that children with HIV disease face barriers to receipt of their HIV medications at school due to intensive control of medications. Another participant described a child with a colostomy bag who was bullied because of their condition. Participants felt that teachers could bridge understanding of children’s PC needs in schools. Another participant suggested that community volunteers could provide support to parents sent home from the hospital with an infant who was not expected to survive. Thus, there appeared to be opportunities to evaluate existing models and potentially create new models for how CHVs and volunteers could support patients with a broader range of palliative needs.

### Evaluation of costs and benefits of PC

Participants pointed to the importance of conducting local cost evaluation of PC. As one stated:

“It’s important for us really to come up with research to quantify how much it costs to provide quality PC for that one patient because to me that is what will inform the decision for planning, from the county levels to the national levels.”

Other participants suggested generating evidence for different models of care, such as home or hospice-based PC vs. standard hospital-based services. Local evidence about cost and benefits of PC was seen as critical to enhance national and external donor support.

### Utility of treatment approaches, including CAM therapies and use of advanced diagnostics at end of life

#### Use of CAM Therapies

Participants expressed interest in exploring incorporation of local symptom management practices, as well as CAM techniques (medicines and medical practices that are not part of standard medical care) including herbal medicines, traditional medicines, yoga, music therapy, message, acupuncture, reflexology, aromatherapy and therapy animals. They endorsed the desire to examine the efficacy, feasibility, and acceptability of such practices in Kenya, asking questions such as: “Are there other remedies that are locally and culturally acceptable?”, “Is it necessary? Would it change a PC patient?” and “Can we replicate (what others have done) at our hospices?”.

#### Use of Advanced Diagnostics and Treatments in Patients with Incurable Disease

Participants also wanted to explore whether there was any benefit to intensive diagnostics and treatment for patients at the end of life. As one participant explained:

“Doctors are subjecting patients to so many investigations which are very expensive and they will not be of any benefit. I have interjected and told the doctor, ‘Give me the justification of doing this investigation; what are the benefits to the patient…if you have no justification, the patient will not go.’”

In regard to use of palliative chemotherapy for patients with incurable cancer, one participant added:

“I would like us to do research on whether it’s really helping, or can we just give morphine, psychosocial support, support the patient instead of…because from my 10 years’ experience, I have really not seen it working; instead palliative chemotherapy just drains somebody and then the patient dies with so much side effects.”

Thus, there was a desire to determine what common medical practices at end of life may be futile and should be avoided, and what common or new medical practices may benefit patients.

### Other suggestions for PC research

Other research questions posed by participants focused on evaluation of strategies for PC advocacy, quality of care and process evaluation, patient-centered care, effect of disclosure of terminal disease status on patient, spirituality in PC, effect of PC on quality of life, and epidemiology research.

### Populations in need of PC research

Participants described three priority populations for PC research: pediatric populations, rural and remote communities, and among patients with conditions requiring PC beyond HIV/AIDS and cancer. Suggestions were made to evaluate and optimize models of pediatric PC and to explore new innovations, such as child life programs that focus on improving quality of life among children through art, play and other strategies to engage children. Participants were interested in establishing recommendations for navigating family dynamics in the treatment of pediatric patients, given the emotional challenges faced by parents when children require PC. They recommended developing new pediatric models to work with new partners in settings such as schools and neonatal units to reach children with special needs who are often overlooked for services in traditional PC settings.

Rural populations were also recognized as having less access to PC and requiring innovative interventions to address care barriers. One participant said:

“In Kenya there are some very hard to reach communities or very hard to reach areas… needs of people in those specific areas might be different from other areas.”

Another added:

“We still have a long way to go because mainly we have concentrated in the big towns. We need to go to the grassroots; We need to go to the community-based health facilities, the dispensaries, and the health centers.”

Mobile and telehealth models were suggested as promising models to enhance the geographical reach of PC warranting evaluation. There was also interest in expanding palliative research to new populations beyond those with HIV and cancer, to include patients with a broader range of conditions, such as TB, malaria and stroke. These three target populations were considered most urgently needed to inform best PC practices.

### Resources for establishing research

While participants were enthusiastic about pursuing PC research in Kenya, they described resources needed to support this research.

#### Human resources and training

Participants expressed need for more PC staffing, staff training on technical aspects of research, and better staff retention once staff are hired and trained. One participant explained: “I am all alone, we need more staff because you are the same person expected to see all the patients.” Another described: “We want to do research but it’s time-consuming and between taking care of patients and going to conduct research, you prefer taking care of patients.”

Regarding the need for technical research training for people working in PC, one participant described: “I feel that before the research if we are doing it as a country, we need a pre-course on PC research.” Several participants noted that access to research certification training is often limited to larger research intensive institutions, while another participant noted the need for free online and easily accessible research training. Participants emphasized the challenge of staff retention following investment of efforts to hire and train staff, noting that recent trainees frequently choose to move to “green[er] pastures” or get transferred to different specialties by hospital management. Reflecting on palliative providers’ need for research training, one participant explained:

“People are just scared of research, especially in terms of methodology. People just think it’s too much mathematics, too much formulas.”

Participants also suggested the need for increased incentives to encourage research skills training and retain promising PC professionals in the field following investment.

#### Enhancement of Health System Data Collection Infrastructure

Participants expressed frustration in trying to conduct research using messy and incomplete sources of data, highlighting the role of data collection metrics, practices, and storage systems in facilitating or acting as a barrier to research. One participant described this issue stating:

“Ideally, we would have a database for the entire continent, where we can really collect data on PC, but we don’t have such a system… we look at data in pieces here and there and that is posing a challenge.”

Another participant described the challenge faced in conducting research among fragmented systems, stating:

“I tried the other day … this same person is appearing three times and I got confused and I decided, it’s like can we stop because you will not give evidenced-based research.”

They also discussed the need for measurement tools to be validated for use among Kenya’s unique population. One stakeholder framed this question, asking: “Do you copy and paste the assessment tools from the western world to this place?”.

#### Fully Operational Cancer Registry

Participants expressed a need to further develop the national cancer registry to support research. Participants explained that the Kenyan government had initiated development of a cancer registry, including training and provision of cancer registry software, but that this work was still in its infancy. As one stakeholder described:

“It’s true that the government has come to help the hospitals set up cancer centers and registries, the only problem has been that they train personnel, within a few days the personnel is nowhere to be seen. They have even brought the software because they have very good software…but you find the people to work on it becomes a problem.”

Another person added:

“I have been involved in research, but we are having difficulties. We don’t have data; we don’t have a cancer registry. For us to start research, I think the base would be for us to have a cancer registry; some of us have been trained on how to start and then the people we were trained with, they went to look for work somewhere else…I was left alone and this needed team work, so Kenya we are depending on very few centers that have cancer registry, i.e., Nairobi, Eldoret and I think Kisumu, and Kenya is large.”

Thus, Participants believed that having a comprehensive cancer registry and staffing to support it locally was critical to enable PC research in Kenya.

#### Additional funding

Participants' comments about barriers to PC research often circled back to funding. In discussing resources needed, such as staffing, training and research infrastructure, participants often pointed to the dependence of such factors on funding. Financial resources were also seen as influencing the capabilities of PC networks, networks which were viewed by many as being “critical” for connecting change makers and technical stakeholders in PC. Funding was described as the “biggest challenge” of research in the area of PC and sustainable funding seen as “a bit of a dream” at this point in time. While participants pointed to the shift in political will in support of PC in recent years, funding was described as highly dependent, with county and national government having little “obligation” to fund work in PC. Current financing sources were described as contributing to “mainly isolated programs rather than national programing for PC.”

## Discussion

In this study, Kenyan PC providers and leaders provided insight into research needed to inform PC delivery in Kenya. They described a need to characterize cultural beliefs about PC and to evaluate awareness of PC among the public and providers. They also described a need to evaluate PC delivery, health system integration and cost, along with targeted studies to examine the benefits of medical tests and treatments when curative care is not possible, CAM, and CHV models of care. Participants recommended populations for whom more research is needed, including focus on a broader range of patients requiring PC beyond cancer and HIV, rural populations where access to care is limited, and pediatric models of PC. Finally, participants suggested resources that could support PC, including human resources, enhanced data collection and management infrastructure, cancer registry completion, and funding.

Participants described a need to characterize beliefs about PC and build interventions to improve awareness, acceptance and use of PC among both the public and providers, echoing a widely reported need in non-African settings to address misunderstanding of what PC is [14, 15]. This was considered important as utilization of PC hinges on public and provider awareness of available palliative services and positive beliefs about the benefits of PC and there are diverse cultural groups across Kenya that have a variety of beliefs about disease, treatment and PC. This finding mirrors those from other studies in Kenya highlighting the need for palliative and bereavement care to be responsive to patients’ unique cultural norms and preferences, [16, 17] and not simply holistically assess and manage the multiple domains of patient suffering [18]. Thus, rigorous studies are needed to map how cultural beliefs and awareness about PC may vary across Kenya and to design culturally-relevant interventions to improve awareness, acceptance and use of PC.

Participants’ interest in evaluation of PC integration across the Kenyan healthcare system was driven by the government’s decentralization of healthcare delivery to the local county level in 2013 [19] and incremental development of a new national cancer registry [20]. Alongside enthusiasm about the potential for increased local access to care, participants were concerned about a lack of resources to support county-level services and uneven progress across counties with varying resources. They cited an interest in understanding how factors such as local staffing, training opportunities, data infrastructure and funding affect care delivery, especially in vulnerable communities. Concern about adequacy of resources to support county-level health services and evaluation capacity has also been reported in a recent systematic review [21]. From a capacity-building standpoint, participants were eager to build upon the evolving health system integration to ensure that critical resources are embedded within the health system for successful health system implementation and sustainability. Thus, research is needed to identify and address critical gaps in infrastructure for palliative care delivery across different settings in Kenya, which in turn can help to build local national capacity for carrying out rigorous palliative care research.

Participants recommended a need to evaluate models for using CHVs and incorporate other volunteer networks, to facilitate PC. CHVs represent a promising workforce in Kenya; they are frequently embedded in communities and can serve as a point of linkage between patients in communities and healthcare systems, as evidenced during COVID-19 [22]. CHVs are also a highly cost-effective workforce for expanding the reach of PC [23]. In a recent systematic review of CHV interventions, integration of CHVs into PC programs was identified as an innovation requiring investigation in the field of patient navigation [24]. Research in, for example, rural areas outside Kolkata, India, based on work in Kerala, has evaluated the use of CHVs and community volunteers in delivering and expanding home-based PC, with promising results indicating its feasibility [25–29]. Given the different contexts across LMICs, a need exists to identify and evaluate contextually relevant strategies for utilizing CHVs to facilitate delivery of PC in Kenya and other LMICs.

Alongside an interest in identifying current treatment practices that may be futile, participants indicated interest in understanding the benefits of CAM treatments in Kenya, a timely topic. A growing body of research is being conducted in high-resource countries to evaluate interventions such as yoga, meditation and acupuncture, [30] and work will be needed to determine which CAM interventions may be promising to translate for use in Kenya or develop locally. Conversely, participants’ call to evaluate the benefits of intensive diagnostics and treatments in patients with terminal illness is a compelling area for research, as futile clinical services can burden both patients and healthcare systems, without providing clinical benefit.

Participants provided insight about populations for whom more research is needed, including a broader focus on all types of conditions that require PC, remote communities where resources are limited, and pediatric populations. The rationale for focusing on a broader range of conditions is that patients with less common conditions—such as childhood developmental delays, tuberculosis and heart failure—often get overlooked for PC and that advocacy for public policy change to fund and integrate PC into health systems will require, among other things, demonstration of the full scale of need for palliative services [31]. The need for PC in remote settings was driven by the fact that resources for delivery of PC are dramatically lower in rural settings, and it will be important to understand needs and barriers to care to be able to develop effective interventions in remote settings. The need for PC in pediatric populations was driven by the fact that additional partners may be needed to help support access to PC in different settings such as neonatal units in hospitals and schools and the need for psychosocial emphasis to help children learn how to navigate living with palliative conditions such as HIV disease or serious developmental disabilities.

Our results contribute uniquely to a very limited body of evidence on research priorities for palliative care. As described in a recent international systematic review by Hasson et al. (2020), only ten research studies have been published on research priorities in palliative care, with nine out of ten of these studies published in high income settings such as the UK, Ireland, US, Australia, New Zealand and Canada [32]. Reflecting upon this limited research, seven key areas of need for palliative care research were identified, which highlighted the need for research on palliative service models, training and education, continuity of care, access to care, communication, patient preferences and experiences with care, and recognition of the needs and preferences of families and caregivers. A cross-cutting theme identified across these studies was that the voice of the healthcare provider dominates existing research, to the neglect of patients and families [32].

Compounding the limitations of research priorities internationally is the relatively poor state of evidence for palliative care priorities in Africa [33]. Powell et al.’s (2014) isolated attempt to develop a prioritized African palliative care research agenda identified three thematic areas (patients, families and volunteers; health providers; health systems) that embraced: care outcomes and impact, palliative care needs of children, impact of palliative care training on care and practice, and integration of palliative care and antiretroviral therapy services, palliative care needs’ assessment at the micro-, meso- and macro-levels, and integration of palliative care into health systems and educational curricula [34]. While the current study similarly identified the integration of palliative care in medical and educational systems, and the needs and experiences of caregivers, it also highlights the socio-cultural aspects of care provision and receipt, awareness of the nature of palliative care among providers and the public, CHV programs, treatment effectiveness and replication of CAM therapies and the benefit of advanced diagnostics and treatment for incurable disease and, importantly, the need to evaluate the financial costs and benefits of palliative care. The findings not only augment existing research but also localize research areas at the country rather than regional level.

Several limitations and strengths of this study should be considered. The study is based upon results from only three FGs (*n* = 22 participants). However, FGs were conducted as part of the national KEHPCA Conference, which resulted in an opportunity to gather meaningful insight from diverse PC providers and leaders from across Kenya, with robust representation of both rural and urban communities. Utilization of a well-trained focus group facilitator who was known to some of the focus group members contributed to high-quality interaction and comfort with expression of both negative and positive viewpoints by group members. With any qualitative study, there is the potential for bias in interpretation of FG discussions. To minimize this bias, two authors with a thorough understanding of the conceptual underpinnings of palliative care independently created their codebooks, coded data from transcripts, and worked together to agree upon final themes and sub-themes. Additional strategies for reducing bias included having a team of researchers create the qualitative interview guide and use of open-ended and neutrally framed questions to avoid biasing the participant. Finally, as noted above, the voice of care providers in informing a research agenda is explored at the expense of that of patients and their families, a limitation that needs to be addressed going forward.

## Conclusions

Kenyan PC providers and leaders expressed enthusiasm at the prospect of conducting research surrounding PC in their nation, recognizing the influence of research in garnering support and informing care practices in Kenya and other African countries. Participants prioritized research investigation of cultural beliefs affecting care, public and provider awareness, promising PC models, affected populations and assessment of PC integration within Kenya’s health system and government agenda. Despite marked interest in exploring lines of PC inquiry, PC participants identified several factors limiting Kenyan practitioners’ capacity to pursue research goals including availability of trained staff, data collection infrastructure, and dedicated and sustainable funding. Stakeholder perspectives can contribute to the formation of a localized PC research agenda and prioritization of limited resources in enhancement of PC research and practice in Kenya. Promotion of PC knowledge in Kenya can also inform PC efforts in other African nations.

## Data Availability

Data generated during the current study are available from the corresponding author upon reasonable request.

## Abbreviations

CAM: Complementary and Alternative Medicine
CHV: Community Health Volunteer
FG: Focus group
KEHPCA: Kenyan Hospices and Palliative Care Association
LMIC: Low-and middle-income countries
PC: Palliative Care
